# Proteolytic control of the RNA silencing machinery

**DOI:** 10.1093/plcell/koae075

**Published:** 2024-03-08

**Authors:** Pascal Genschik, Marlene Schiaffini, Esther Lechner

**Affiliations:** Institut de Biologie Moléculaire des Plantes, CNRS, Université de Strasbourg, 12, rue du Général Zimmer, Strasbourg 67084, France; Institut de Biologie Moléculaire des Plantes, CNRS, Université de Strasbourg, 12, rue du Général Zimmer, Strasbourg 67084, France; Institut de Biologie Moléculaire des Plantes, CNRS, Université de Strasbourg, 12, rue du Général Zimmer, Strasbourg 67084, France

## Abstract

Studies in plants were often pioneering in the field of RNA silencing and revealed a broad range of small RNA (sRNA) categories. When associated with ARGONAUTE (AGO) proteins, sRNAs play important functions in development, genome integrity, stress responses, and antiviral immunity. Today, most of the protein factors required for the biogenesis of sRNA classes, their amplification through the production of double-stranded RNA, and their function in transcriptional and posttranscriptional regulation have been identified. Nevertheless, and despite the importance of RNA silencing, we still know very little about their posttranslational regulation. This is in stark contrast with studies in metazoans, where different modifications such as prolyl hydroxylation, phosphorylation, sumoylation, ubiquitylation, and others have been reported to alter the activity and stability of key factors, such as AGO proteins. Here, we review current knowledge of how key components of the RNA silencing machinery in plants are regulated during development and by microbial hijacking of endogenous proteases.

## Teaser

We review proteolytic regulation by endogenous proteases and microbial hijacking of key components of the RNA silencing machinery.

## Introduction

The mechanism of RNA silencing is conserved in eukaryotes and has been exhaustively reviewed elsewhere (Ghildiyal and Zamore 2009; Bologna and Voinnet 2014; Treiber et al. 2019). It is essential in controlling gene expression for the proper development of an organism, but it is also key for the control of transposition events and heterochromatin maintenance and, at least in invertebrates and plants, plays a prominent role in defense against viruses. Briefly, the RNA silencing mechanism involves processing of double-stranded (ds)RNA by the RNase III enzyme Dicer into small RNA (sRNA) of 21 to 24 nucleotides (nts) in length. Those sRNAs associate with ARGONAUTE (AGO) proteins to form RNA-induced silencing complexes (RISCs; Meister 2013; Poulsen et al. 2013). The sequence of the sRNA provides the specificity for RISCs to interact with their transcript targets, based on sequence complementarity, resulting in their downregulation.

In plants, 2 broad categories of sRNA have been described (Axtell 2013). The first category consists of microRNA (miRNA), which arises from noncoding *MIRNA* (*MIR*) genes. After processing, miRNAs are loaded into AGO proteins and function posttranscriptionally by repressing the level and/or expression of their mRNA targets. The second category, called small interfering RNA (siRNA), is processed from dsRNAs that originate from different sources, such as transposons, endogenous inverted repeats, viral RNA, or even transgenes. After AGO loading, siRNA also acts as repressor of expression either transcriptionally or posttranscriptionally, by mediating RNA cleavage and/or translational repression. How and where at the tissue level most components of the RNA silencing machinery are produced are overall well understood. Nonetheless, it is also essential to maintain proteins at the appropriate concentration or even eliminate them, once their function is no longer required and could potentially become toxic for the cell. In all eukaryotes, the 2 major pathways for protein degradation are the ubiquitin–proteasome system (UPS) and autophagy. Perturbations in either of these proteolytic pathways are very detrimental for cells. For the sake of clarity, we will briefly introduce both pathways below.

The UPS is likely the fastest and most selective way to degrade proteins (Ciechanover et al. 2000; Vierstra 2009). In this pathway, ubiquitin (Ub), a peptide of 76 amino acids, is covalently attached to the protein substrate to be degraded. This is achieved through an enzymatic cascade in which Ub ligases (also known as E3 enzymes), together with Ub-conjugating enzymes (E2s), confer the substrate specificity of the ubiquitylation reaction (Zheng and Shabek 2017). In fact, E3 Ub ligases need to recognize a minimal structural element within a protein target, called a “degron,” which is sufficient for its degradation. Notably, Ub can itself be ubiquitylated on any of its lysine residues generating often complex polyubiquitin chains with different topologies (Kwon and Ciechanover 2017). E3 Ub ligases belong to different conserved classes and in plants, include monomeric RING (really interesting new gene), HECT (homologous to the E6AP carboxyl terminus), the APC/C (anaphase-promoting complex/cyclosome), and the largest group defined as CULLIN-RING LIGASES (CRLs; Hua and Vierstra 2011). CRLs are multimeric E3s, in which 1 particular protein called CULLIN serves as a molecular scaffold linking up the catalytic module, composed of the RING finger protein RBX1 (RING BOX PROTEIN1) and a Ub-conjugating E2 enzyme, to a specific substrate recognition module, which physically interacts with target proteins. Depending on the CULLIN protein, CRLs are subdivided into different families, including the SCF, CRL3, and CRL4 type of E3 Ub ligases (Hua and Vierstra 2011). Target recognition is ensured by F-box proteins (they are roughly 700 hundreds in Arabidopsis), BTB (bric-a-brac–tramtrack–broad complex), and DCAF (DDB1-CUL4-ASSOCIATED FACTOR) proteins for SCF, CRL3, and CRL4 complexes, respectively. Once a protein is ubiquitylated, and depending on the topology of the Ub chain, it can be directed to the proteasome where it will be unfolded and degraded (Bard et al. 2018).

The second proteolytic pathway, referred to as autophagy, requires dozens of proteins annotated as autophagy-related proteins (ATGs; Kroemer et al. 2010; Mizushima et al. 2011; Marshall and Vierstra 2018). Proteins to be degraded, as well as other cellular contents (called cargos), are captured within a double-membrane vesicle termed the phagophore, mostly emanating from the endoplasmic reticulum (ER), which then seals to form an autophagosome that delivers cargos to lytic compartments (lysosomes in animals or the vacuoles in plants). Under starvation and other stresses, autophagy acts in a nonselective way to degrade proteins and other cargos in bulk, which provides metabolic building blocks and energy to cells. However, autophagy can also act in a more selective manner, to remove some organelles or even target specifically some proteins (Rogov et al. 2014; Khaminets et al. 2016). To do so, selective autophagy employs specialized cargo receptors that anchor the cargo to autophagy machinery and in particular interact with ATG8 proteins, which decorate the autophagosome membranes and are involved in their maturation and fusion with the lytic compartment. Notably, ubiquitylation is an indispensable signal to initiate some types of selective autophagy. In plants, however, only a few selective autophagy receptors have been identified, and the role played by ubiquitylation in selective autophagy is still not well understood (Michaeli et al. 2016; Stephani and Dagdas 2020).

## The core plant Microprocessor machinery

In plant cells, miRNA biogenesis occurs exclusively in the nucleus. *MIR* genes are transcribed by RNA polymerase II (Pol II) and form hairpin-like structures called primary miRNAs (pri-miRNAs), which for a large portion are processed cotranscriptionally by the RNase type III enzyme DICER-LIKE 1 (DCL1) to form miRNA/miRNA* duplexes (reviewed in Mencia et al. 2023). DCL1 does not operate alone and requires the zinc finger protein SERRATE (SE), and the dsRNA-binding protein HYPONASTIC LEAVES 1 (HYL1), along with other accessory proteins, forms the Microprocessor (Bologna and Voinnet 2014). As miRNA/miRNA* duplexes contain hydroxyl groups at the 3′ ends, they need to be methylated by the methylase HUA ENHANCER 1 (HEN1), which protects them from subsequent degradation (Yu 2005).

**Figure 1.**
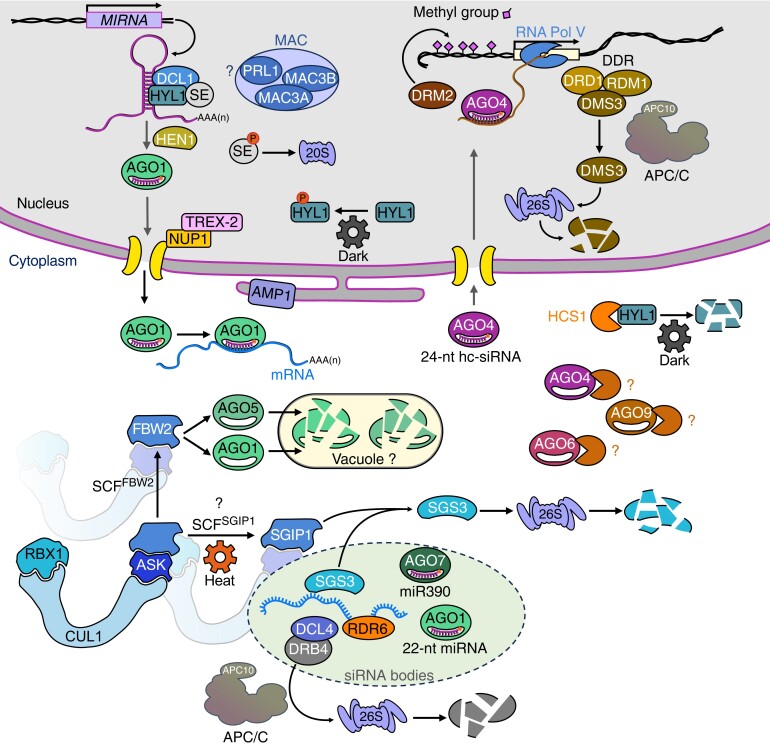
Schematic representation of posttranslational regulations by proteolysis of components of the RNA silencing machinery. Nuclear and cytoplasmic compartments are indicated. The FBW2-mediated degradation of AGO1 in the vacuole and the protease(s) degrading AGO4/4/9 are still unknown and indicated by question marks. Note that for SCF-type Ub E3 ligases, the name of the F-box is indicated in superscript.

Several studies reported that HYL1 is subjected to proteolysis, affecting the Microprocessor activity and thus miRNA biogenesis. By treating Arabidopsis seedlings with cycloheximide (CHX), which blocks protein synthesis, it was first shown that HYL1 is a short-lived protein (Cho et al. 2014). Interestingly, the turnover of HYL1 is dependent on light conditions. In the dark, HYL1 is degraded (Fig. 1), while in the light, it is stabilized by a mechanism involving the E3 Ub ligase CONSTITUTIVE PHOTOMORPHOGENIC 1 (COP1). Consistently, it was further shown that miRNA biogenesis gradually decreased during prolonged darkness but was recovered upon light treatment (Achkar et al. 2018). Moreover, the stability of HYL1 depends on its phosphorylation. Thus, it was shown that HYL1 is a substrate of SnRK2 kinases and that its abundance in an *snrk2.2/3/6* triple mutant was significantly reduced (Yan et al. 2017). A reduced amount of HYL1 protein was also observed when seedlings of the *snrk2* multiple mutants were subjected to osmotic stress, suggesting that different environmental conditions could impact HYL1 protein homeostasis (Yan et al. 2017). Conversely, the C-TERMINAL DOMAIN PHOSPHATASE-LIKE 1 and 2 (CPL1 and CPL2) dephosphorylate HYL1 (Manavella et al. 2012) and, in a *cpl1/cpl2* double mutant, HYL1 is more resistant to degradation in the dark (Achkar et al. 2018). Mechanistically, it was proposed that in the dark, the nonphosphorylated HYL1 form, which is also the active form of the protein, is degraded in the cytosol (Fig. 1), but that a nuclear reserve pool of phosphorylated HYL1 remains resistant to degradation (Achkar et al. 2018). Upon light treatment, HYL1 would be dephosphorylated from this reserve pool to restore miRNA production. In addition, the plant-specific ESCRT component, FYVE domain protein required for endosomal sorting 1 (FREE1), interacts with CPL1 in the nucleus affecting the phosphorylation of HYL1 (Li et al. 2023). Thus, *FREE1* acts as a repressor of miRNA biogenesis, and its inactivation increases the active hypophosphorylated, but also unstable, pool of HYL1. Another study however reported that the PP4 (PROTEIN PHOSPHATASE 4) and SMEK1 (SUPPRESSOR OF MEK 1) complex also dephosphorylates HYL1, but this time, dephosphorylation would increase HYL1 stability (Su et al. 2017). Further work will be required to determine the exact role of phosphorylation for HYL1 turnover. Regardless of the status of HYL1 phosphorylation, an important question remained: which is the protease that eliminates HYL1 in darkness. Recent work by Jung et al. (2022) identified HYL1-CLEAVAGE SUBTILASE 1 (HCS1) as a cytoplasmic protease targeting HYL1 for degradation (Fig. 1 and Table 1). This study also shed new light on the possible function of COP1 in protecting HYL1 from degradation. Hence, it was shown that light triggers the cytoplasmic localization of COP1, which inhibits HCS1 via direct protein–protein interaction protecting HYL1. In the dark, COP1 moves to the nucleus allowing HCS1 to degrade HYL1.

**Table 1.** Summary of RNA silencing proteins known to be selectively degraded under normal (bold) and upon viral hijacking (italics) conditions. “?” stands for unknown.

Proteins	Degrons	Ubi ligases	Proteases	Contexts	References
**HYL1**	**Phospho-dependent**	**No**	**HCS1**	**Dark, osmotic stress**	Cho et al. 2014; Yan et al. 2017; Achkar et al. 2018; Su et al. 2017; Jung et al. 2022
**DCL1**	**ND**	**No**	**20S proteasome**	**Dark**	Choi et al. 2020
**SE**	**Phospho-dependent**	**No**	**20S proteasome**	**ND**	Li et al. 2020
**AGO1**	**ND**	**SCFFBW2**	**ND**	**Avoid spurious loading of sRNA**	Earley et al. 2010; Ré et al. 2020; Hacquard et al. 2022
**AGO2**	**RG/GR repeat methylation?**	**?**	**Proteasome**	**Plant immune responses**	Hu et al. 2019
**AGO5**	**ND**	**SCFFBW2**	**ND**	**ND**	Hacquard et al. 2022
**AGO4/6/9**	**ND**	**?**	**?**	**Lack of hc-siRNA**	Li et al. 2006; Havecker et al. 2010
**SGS3**	**ND**	**SGIP1**	**Proteasome**	**Heat stress**	Zhong et al. 2013; Liu et al. 2019
**DRB4**	**ND**	**APC/C**	**Proteasome**	**Homeostasis**	Marrocco et al. 2012
**DMS3**	**D-box**	**APC/C**	**Proteasome**	**DDR complex stoichiometry, cell cycle**	Zhong et al. 2019
*AGO1 and other AGOs*	*Motif in the DUF1785*	*SCF^P0^ poleroviruses*	*Autophagy* (*ATI1/2*)	*Suppression of RNAi*	Baumberger et al. 2007; Bortolamiol et al. 2007; Csorba et al. 2010; Derrien et al. 2012; Michaeli et al. 2019
*AGO1 and AGO2*	*ND*	*P25 potexviruses*	*Proteasome*	*Suppression of RNAi*	Chiu et al. 2010
*SGS3*	*ND*	*VPg potyviruses*	*Autophagy and proteasome*	*Suppression of RNAi*	Cheng and Wang 2017

Less is understood regarding the turnover of other components of the Microprocessor. For instance, the steady-state level of the DCL1 protein is also low in dark-grown seedlings, but accumulates to higher levels after pharmacological inhibition of the proteasome by MG132 (Choi et al. 2020). An intriguing study also reported that SE physically binds PAG1, which is a subunit of the 20S proteasome (Li et al. 2020) and is degraded in a Ub-independent manner (Fig. 1 and Table 1). Hence, SE, which is a short-lived protein, is stabilized in mutants of the 20S proteasome or after MG132 treatment, but curiously not by pharmacological inhibition of the Ub-activating enzyme E1 using the drug PYR-41, suggesting that its turnover does not depend on Ub. The N-terminal region of SE is disordered and unstructured, and the authors speculate that the proteasome could directly eliminate the excess of SE, but not when it is assembled and protected in macromolecular complexes (Li et al. 2020). Notably, the intrinsically disordered region located in its N-terminal region of SE was also shown to undergo phase separation, which is important for the formation of dicing bodies (D-bodies) where miRNA biogenesis occurs (Xie et al. 2021). As for HYL1, phosphorylation also impinges SE stability. Thus, it was revealed that SE is phosphorylated by the pre-mRNA processing 4 kinase A (PRP4KA), which potentially triggers its degradation by the 20S proteasome (Wang et al. 2022). A recent study implicated 2 intrinsically disordered proteins, called SAID1/2, in the PRP4KA-dependent phosphorylation of SE and its degradation, likely by hijacking pri-miRNAs from SE, but the physiological significance of this regulation is still unclear (Shang et al. 2023). Also, whether a phosphatase could revert the action of PRP4KA to stabilize SE, where at the subcellular level does SE degradation occur, and what are the physiological contexts of its proteolysis are still open questions. Last but not least, it has been shown that the conserved MOS4-associated complex (MAC) binds components of the Microprocessor and positively regulates miRNA and siRNA accumulation (Zhang et al. 2014; Li et al. 2018). One of its components, PRL1, is a DCAF adaptor protein of CRL4 E3 Ub ligases (Lee et al. 2008; Fonseca and Rubio 2019), while 2 other subunits, MAC3A and MAC3B, are conserved U-box-containing proteins with demonstrated Ub ligase activity at least in vitro (Li et al. 2018). How the MAC directly or indirectly enhances miRNA biogenesis is still unclear (Fig. 1), but the protein levels of SE and DCL1 were not changed in a *mac3a mac3b* double mutant, suggesting that these proteins are not direct substrates. Thus, it is worth to further investigate how MAC ubiquitylation activity modulates sRNA biogenesis. Finally, still enigmatic is the role played by the F-box gene *HAWAIIAN SKIRT* (*HWS*) in miRNA biogenesis, degradation, or action. Mutation of *HWS* increases the steady-state levels of several miRNAs, while its overexpression leads to an opposite phenotype and *hws* mutants were shown to resemble known miRNA mutants, such as *hyl1-2* or *se-3* (Lang et al. 2018). It is possible that HWS also acts downstream of miRNA biogenesis, as it could be involved in the clearance of RISCs associated with mimicry targets, though at present its substrate(s) remain unknown (Lang et al. 2018; Mei et al. 2019).

## Regulation of AGO1-RISC

Downstream of the Microprocessor, AGO proteins form RISCs (Poulsen et al. 2013). Among the 10 AGOs encoded by the Arabidopsis genome, AGO1 plays a central role in both miRNA- and siRNA-directed silencing pathways (Mi et al. 2008). The assembly of the AGO1-miRNA RISCs occurs mainly in the nucleus, but they are subsequently exported to the cytoplasm via a nuclear export signal in AGO1 (Bologna et al. 2018; Fig. 1). When loaded with miRNA on the ER, AGO1 mediates endonucleolytic cleavage and translation repression of target transcripts (Baumberger and Baulcombe 2005; Brodersen et al. 2008; Li et al. 2013). To maintain AGO1 protein homeostasis, excessive AGO1 protein accumulation is repressed by a feedback loop involving miRNA168, which targets *AGO1* transcript (Mallory and Vaucheret 2010). This feedback loop is instrumental as the expression of a miR168-resistant *AGO1* mRNA induces severe development defects in Arabidopsis due to AGO1 overaccumulation (Vaucheret et al. 2004). This may raise a question about the necessity to elaborate another mechanism beside the miR168 feedback loop to restrain AGO1 protein accumulation.

Indeed, it has been shown that the stability of Arabidopsis AGO1 protein is correlated with its loading state. Hence, different mutations affecting sRNA biogenesis and their availability lead to AGO1 degradation (Derrien et al. 2012). Degradation of unloaded AGOs is also conserved in mammals and flies (Martinez and Gregory 2013; Smibert et al. 2013). The first hint to the mechanism of AGO1 turnover came from a genetic suppressor screen of a null allele of *SQUINT* (*SQN*), encoding a cyclophilin-40 chaperone that acts as a positive regulator of AGO1 activity. This screen revealed an F-box protein called F-BOX WITH WD-40 2 (FBW2), which mutation increases the abundance of AGO1 (Earley et al. 2010). Note that FBW2 has been misannotated and does not contain a WD40 domain, but instead comprises, in addition to its F-box, LRR repeats commonly found in plant F-box proteins (Gagne et al. 2002) and an unstructured C-terminal domain. FBW2 interacts with core components of the SCF such as ARABIDOPSIS SKP1-LIKE1 (ASK1), the RING-BOX PROTEIN1 (RBX1), and CULLIN1 (CUL1) both in yeast and in planta (Risseeuw et al. 2003; Hacquard et al. 2022), indicating that it is part of an SCF complex (hereafter called SCF^FBW2^; Fig. 1 and Table 1). In transgenic Arabidopsis lines overexpressing FBW2 and in *fbw2* null mutants, AGO1 protein steady-state level was found reduced and slightly increased, respectively, without affecting the *AGO1* transcript level, thus supporting a role of FBW2 to maintain AGO1 protein homeostasis under normal growing conditions (Earley et al. 2010; Hacquard et al. 2022). The fact that the *fbw2* mutation restored AGO1 protein levels in various mutants affecting sRNA biogenesis, but also mutations affecting AGO1 loading, such as *ago1-42*, as well as other observations not discussed here, argues that FBW2 targets preferably the unloaded form of AGO1 (Hacquard et al. 2022; Fig. 1). Likewise, FBW2 promotes the degradation of AGO5, which belongs to the same phylogenetic clade as AGO1, but not of AGO2 or AGO4 belonging to different clades. How FBW2 discriminates among the AGO substrates is still unclear, and a direct interaction of FBW2 with AGO proteins has not yet been demonstrated. Interestingly, the C-terminal domain of FBW2 contains tryptophan repeats reminiscent of a putative AGO hook motif (Till et al. 2007), so additional work is required to understand the structural determinants of this interaction. Another aspect that remains obscure is by which proteolytic machinery FBW2 mediates AGO1 degradation and which type of Ub chains is involved in this process. It was previously reported that the proteasome inhibitor MG132 was unable to restore AGO1 protein levels in *FBW2* overexpressing plants (Earley et al. 2010). It is also noteworthy that the N domain of AGO1 interacts with the autophagy cargo receptors ATG8-INTERACTING PROTEINS 1 AND 2 (ATI1 and ATI2; Bressendorff et al. 2023), and that *ati1/2* double mutant plants showed a mild increase in AGO1 protein level significantly enhancing sense transgene-mediated posttranscriptional gene silencing (S-PTGS) activity (Michaeli et al. 2019). Whether FBW2 triggers AGO1 degradation by selective autophagy and which autophagy receptor(s) is/are involved are interesting questions to solve in the future (Fig. 1).

Why is it necessary to degrade unloaded AGO1 is another intriguing question since under standard growing conditions, *fbw2* mutant plants exhibit no visible phenotype (Earley et al. 2010; Hacquard et al. 2022). A clue to this question came from the observation that in plants lacking *FBW2* and which are impaired in sRNA accumulation (such as *hyl1* or *hen1* mutants), AGO1 stabilization further worsens their phenotype (Hacquard et al. 2022). Hence, it was shown that nondegradable AGO1 protein binds illegitimate sRNA, leading to the cleavage of different target genes. Therefore, FBW2 degradation is important to avoid AGO1 spurious loading of sRNA, which could conditionally become detrimental for cells. Notably, FBW2 may also turnover certain forms of loaded AGO1. For instance, a recent report highlighted that AGO1 is degraded when it is bound to an mRNA target under prolonged conditions, such as in the presence of noncleavable artificial miRNA target mimics (Ré et al. 2020). This is in line with the observation that FBW2 also associates with membrane-bound AGO1, which is expected to be loaded and bound to its target RNA (Hacquard et al. 2022). It will be interesting to clarify whether the binding of AGO1 to its target would expose the same degron as unloaded AGO1 allowing its recognition by FBW2. Finally, *FBW2* is transcriptionally repressed by CURLY LEAF (CLF), encoding a subunit of the polycomb repressor complex 2 (PRC2), and it was shown that this regulation may be important for AGO1 protein homeostasis when plants are exposed to UV radiation (Ré et al. 2020). Thus, it will be worth to further investigate the FBW2-mediated AGO1 degradation pathway in the context of abiotic and biotic stresses, as such conditions are known to foster RISC reprogramming allowing plants to respond to stress.

## Endogenous secondary siRNA biogenesis

SiRNAs are generated through the cleavage of dsRNA, and they fall into different subclasses (Axtell 2013; Pumplin and Voinnet 2013; Zhan and Meyers 2023). Two proteins, SUPPRESSOR OF GENE SILENCING 3 (SGS3) and RNA-DEPENDENT RNA POLYMERASE 6 (RDR6) are essential for the process of dsRNA synthesis, and they largely contribute to the production of secondary endogenous siRNA such as phasiRNAs and also viral siRNAs (vsiRNAs; Pumplin and Voinnet 2013; Borges and Martienssen 2015; Liu et al. 2020). How SGS3 is recruited to its target transcripts is not fully understood, but it seems to require a conformational change of 22-nt miRNA-AGO1-RISC or miR390-AGO7-RISC bound to their targets (Fei et al. 2015; Liu et al. 2020), causing ribosome stalling to stimulate secondary siRNA production (Iwakawa et al. 2021). The resulting dsRNA is subsequently cleaved by different DCLs and in particular DCL4, which, assisted by DOUBLE-STRANDED RNA BINDING FACTOR 4 (DRB4), produces 21-nt phasiRNAs (reviewed in Liu et al. 2020). Moreover, the whole process of secondary siRNA production apparently happens in membraneless cytosolic structures called siRNA bodies (Jouannet et al. 2012; Fig. 1).

Interestingly, it has been shown that warm temperatures inhibit siRNA biogenesis in Arabidopsis, and this was correlated with a decrease in the steady-state level of an SGS3-GFP reporter protein (Zhong et al. 2013). The heat-induced degradation of SGS3 was found attenuated in the presence of the proteasome inhibitor MG132 and a screen of heat-responsive Ub E3 ligases identified SGS3-INTERACTING PROTEIN 1 (SGIP1) as a possible candidate to trigger this degradation (Liu et al. 2019; Fig. 1 and Table 1). Indeed, SGIP1 is an F-box protein, which directly interacts and ubiquitylates SGS3, and its overexpression leads to reduced levels of SGS3 as well as trans-acting siRNAs (tasiRNAs). Interestingly, SGIP1 seems to be part of an epigenetic regulatory network to confer transgenerational stress adaptation in plants (Liu et al. 2019). Nonetheless, some points will need further clarifications. As SGS3 has been reported to undergo liquid–liquid phase separation (LLPS) and to be critical for the formation of cytoplasmic siRNA bodies during stress (Kim et al. 2021; Tan et al. 2023), one may wonder why this protein is degraded and also how SGIP1 would have access to it in these condensates. AGO1 also undergoes LLPS after a short heat stress treatment and colocalizes with SGS3; however, the AGO1 protein is not degraded, but its amount rather increases (Blagojevic et al. 2024). Downstream of dsRNA production, DRB4, a cofactor of DCL4, is also degraded by the proteasome (Marrocco et al. 2012). The Ub E3 ligase involved in this mechanism is the multimeric APC/C, and it was shown that DRB4 specifically interacts with one of its core subunits APC10 (Fig. 1 and Table 1). While the DRB4 protein overaccumulates in Arabidopsis *APC/C* hypomorphic mutant lines, this nevertheless did not affect dramatically siRNA production, raising the question of the physiological role of this regulation. Finally, it was reported that the 26S proteasome subunit RPT2a promotes the production of transgene-derived siRNA and thus posttranscriptional gene silencing (PTGS) activity (Kim et al. 2019). However, this effect was explained by the proteasomal regulation of a subset of RNA quality control (RQC) components rather than components of PTGS.

## Regulation of RNA-directed DNA methylation

Another important class of sRNA is heterochromatic siRNAs (hc-siRNAs; Borges and Martienssen 2015; Rymen et al. 2020; Zhan and Meyers 2023). These hc-siRNAs are involved in the RNA-directed DNA methylation (RdDM) pathway to mediate the transcriptional gene silencing (TGS) of transposable elements (TEs) and other DNA sequences. The biogenesis of hc-siRNAs depends on the plant–specific RNA polymerase Pol IV, which transcripts, originating from silenced TEs and tandem repeats, are processed into dsRNAs by RDR2. This nuclear dsRNA is further processed by DCL3 into 24–nt-long hc-siRNAs that are presumably exported in the cytoplasm where they preferentially bind to AGO4 and also its closely related AGO6 and AGO9 proteins (Matzke and Mosher 2014; Liu et al. 2022). Note that besides the production of 24-nt siRNAs by Pol IV–RDR2–DCL3, some other pathways referred to as noncanonical RdDM mechanisms have been described (Cuerda-Gil and Slotkin 2016). AGO4-hc-siRNA complexes return back to the nucleus where they bind to complementary sequences in nascent transcripts of another plant–specific RNA polymerase Pol V leading to the recruitment of DOMAINS REARRANGED METHYLTRANSFERASE 2 (DRM2), which catalyzes de novo methylation of cytosines on chromatin (Borges and Martienssen 2015; Fig. 1). Note that the recruitment of Pol V to its target genes also requires other components such as the so-called DDR complex (DRD1, DMS3, and RDM1; Zhong et al. 2012 and references therein).

As for AGO1, it appears that the loading of AGO4 by sRNA is essential for its stability. Indeed, mutations of genes encoding the Pol IV subunit *NRPD1a* or genes required for the production of hc-siRNAs, such as *RDR2* and *DCL3*, all exhibit decreased accumulation of AGO4 protein (Li et al. 2006). In fact, the whole AGO4 subclade including AGO6 and AGO9 were found to be unstable in the absence of 24-nt hc-siRNA (Havecker et al. 2010). In addition, AGO4 forms also a complex with the DNA repair factor DNA DAMAGE BINDING2 (DDB2) involved in DNA methylation, even in the absence of DNA damage (Schalk et al. 2016). Interestingly, *DDB2*-deficient plants as well showed a drop of AGO4 protein content, though the mechanism by which this occurs is unknown. Nevertheless, the SCF^FBW2^ that targets unloaded AGO1 was unable to destabilize AGO4 in Arabidopsis (Hacquard et al. 2022) excluding a role of this Ub E3 ligase in this process. At present, the proteolytic mechanism by how the AGO4/6/9 group is destabilized remains puzzling (Fig. 1 and Table 1). Performing a forward genetic screen to identify mutants stabilizing AGO4 in one of the hc-siRNA biogenesis-deficient backgrounds might provide some clue to elucidate this mechanism.

Interestingly, a recent study reported that a number of TEs were derepressed in mutants of the APC/C Ub E3 ligase (Zhong et al. 2019). By using different protein interaction assays, these authors could show that DMS3, a protein of the DDR complex, interacts with the APC10 subunit leading to its ubiquitylation by the APC/C and its subsequent degradation by the proteasome (Fig. 1 and Table 1). It was proposed that the APC/C contributes to maintain the stoichiometric balance of the DDR complex. Strikingly, DMS3 protein level fluctuates during the cell cycle suggesting an important role for the APC/C to delimit RdDM activity during cell cycle progression (Zhong et al. 2019).

Finally, as stressful conditions, such as heat, can lead to the activation of some transposons (Dubin et al. 2018; Ito 2022), presumably to help plants to adapt to their environment, it might be interesting to investigate the role of selective proteolysis in this process. Hence, in such a hypothetic scenario, stress-induced degradation of proteins involved in DNA methylation pathways, including those involved in RdDM, might provide a mechanism for TE reactivation.

## Manipulation of RNA silencing by microbial proteolytic hijacking

**Figure 2.**
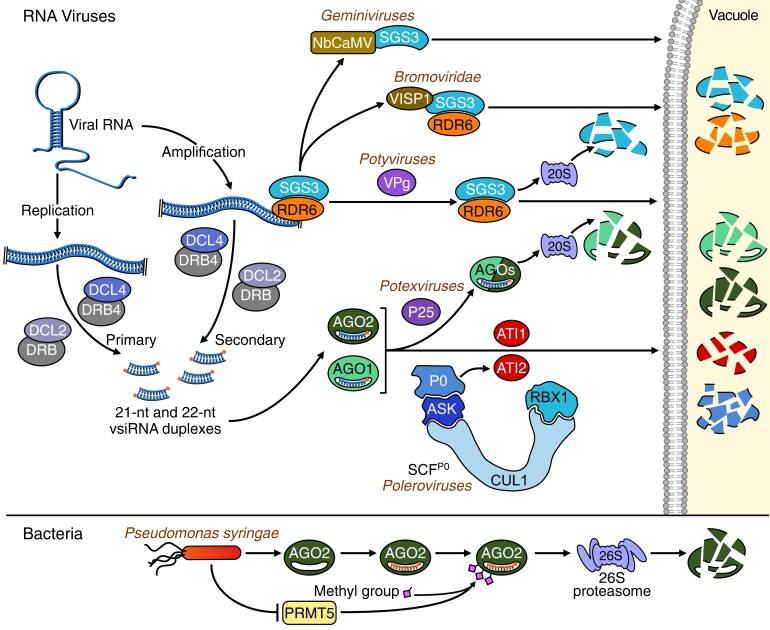
Overview of microbial-mediated degradation pathways of RNA silencing proteins.

RNA silencing is at the forefront of antiviral immunity in plant cells (Pumplin and Voinnet 2013; Yang and Li 2018). This mechanism is particularly efficient against RNA viruses, whose dsRNA produced during viral replication is recognized and processed by DCL4, DCL2, and to a lesser extent DCL3 and their associated DRB cofactors to produce, respectively, 21-, 22-, and 24-nt vsiRNA duplexes (Fig. 2). These vsiRNA duplexes are then unwound and loaded into AGO proteins to form vsiRNA-RISCs. AGO2 seems to play a major antiviral role, alongside AGO1, AGO5, and other AGOs (Carbonell and Carrington 2015). Antiviral vsiRNA-RISCs target, in a sequence-specific manner, the viral RNA leading to its endonucleolytic cleavage and/or translational repression. Moreover, this mechanism can be further amplified through the conversion of viral single-stranded (ss)RNA into dsRNA by the host RDR6, together with SGS3 and SDE5, which upon cleavage by DCLs produces secondary vsiRNAs (Fig. 2).

To counter this highly efficient antiviral immune mechanism, viruses acquired various viral suppressors of RNA silencing (VSRs) able to target different steps of the pathway (Pumplin and Voinnet 2013; Csorba et al. 2015), though we will discuss here only VSRs connected to proteolysis. One of them, which has been extensively characterized over the last years, is the P0 protein of phloem-restricted poleroviruses, such as *Turnip yellows virus* (TuYV). It was found that the P0 VSR encodes an F-box protein that hijacks the host Ub pathway by assembling an SCF Ub E3 ligase to promote the degradation of AGO1 (Pazhouhandeh et al. 2006; Baumberger et al. 2007; Bortolamiol et al. 2007; Csorba et al. 2010; Fig. 2 and Table 1). While P0-mediated degradation of AGO1 was insensitive to inhibition of the proteasome (Baumberger et al. 2007), it was found that AGO1 degradation occurs in the vacuole and can be blocked by the inhibition of autophagy (Derrien et al. 2012). In fact, P0 and AGO1 associate on the ER and are both delivered to the vacuole with the help of ATI1 and ATI2, 2 plant specialized autophagy cargo receptors (Michaeli et al. 2019; Fig. 2). Furthermore, the degron allowing AGO1 to be recognized by P0 is conserved and confers P0-mediated degradation to other AGO proteins (Derrien et al. 2018; Trolet et al. 2019), thus making P0 particularly effective as a VSR. However, during genuine TuYV infection, AGO1 was found unexpectedly stabilized rather than degraded in the vasculature tissue of Arabidopsis plants (Clavel et al. 2021). This suggests that in a viral context, P0 may only destabilize a small pool of AGO1, likely at the site of TuYV replication, and does not spread throughout cells. Other VSRs can also target AGO proteins for degradation. This is the case for the P25 protein encoded by Potato Virus X (PVX), which interacts with AGO1 and AGO2, but not AGO5, and promotes at least AGO1 degradation in a proteasome-dependent manner when transiently expressed in *Nicotiana benthamiana* leaves (Chiu et al. 2010; Fig. 2 and Table 1). P25 has no hallmarks of Ub E3 ligases, and its mode of action still remains unclear. A P25-related VSR called TGBp1 of *Plantago asiatica mosaic virus* (PlAMV) does not seem to act at the level of AGO1, but rather inhibits dsRNA synthesis by interacting and forming aggregates with RDR6 and SGS3 (Okano et al. 2014). Another VSR, called viral genome-linked protein (VPg) of *Turnip mosaic virus* (TuMV) interacts with SGS3 and promotes its degradation by both the autophagy and the Ub–proteasome pathways (Cheng and Wang 2017). In this case, both SGS3 and its partner protein RDR6 are codegraded to presumably enhance host susceptibility to the virus (Fig. 2 and Table 1). Note that TuMV encodes additionally another VSR, HC-Pro, which functions through the sequestration of vsiRNAs and is far more efficient in suppressing host RNA silencing. Thus, the reason why TuMV elaborated a second layer of counter defense involving proteolysis is also not clear.

Markedly, viruses also manipulate endogenous host proteins to promote the degradation of silencing components. An interesting recent study highlighted that *Cucumber mosaic virus* (CMV) infection in Arabidopsis induces the expression of a small peptide composed of 71 amino acids, called VISP1, carrying a Ub-interacting motif (UIM) able to interact with ATG8. Hence, it was shown that VISP1 serves as an autophagy cargo receptor and triggers autophagic degradation of SGS3/RDR6 bodies to negatively regulate siRNA amplification (Tong et al. 2021). DNA viruses as well are able to use such tricks. For instance, during infection of *N. benthamiana* by geminiviruses, it was shown that a host calmodulin-like protein is mobilized and acts as an endogenous RNA silencing suppressor by promoting once again SGS3 degradation most likely through the autophagy pathway (Li et al. 2017). Note however that while autophagy can be manipulated by several viruses to counteract antiviral silencing, it also serves as a component of antiviral defense by degrading viral proteins and in particular by eliminating a number of different VSRs (Clavel et al. 2017; Kushwaha et al. 2019; Tong et al. 2023).

Finally, RNA silencing plays also an important function in host immune responses against nonviral plant pathogens, and the induction or repression of specific miRNAs contributes to defense responses (Pumplin and Voinnet 2013; Song et al. 2019). For instance, the expression of Arabidopsis AGO2 was shown to be highly induced by *Pseudomonas syringae* (Pst), and *ago2* mutants are more susceptible to both virulent and avirulent strains of Pst (Zhang et al. 2011). Indeed, AGO2 bound to miR393* negatively regulates the expression of a SNARE protein gene leading to increased secretion of antimicrobial peptides. Interestingly, it was recently shown that AGO2 is also regulated at the posttranslational level. Hence, AGO2 N-terminal domain, enriched with arginine–glycine RG/GR repeats, is methylated by PROTEIN ARGININE METHYLTRANSFERASE 5 (PRMT5; Hu et al. 2019). RMT5-mediated arginine methylation inhibits AG02-mediated silencing by 2 mechanisms: (i) promoting AGO2 protein degradation by the proteasome (Fig. 2 and Table 1) and (ii) recruiting of 2 Tudor-like proteins, TSN1 and TSN2, able to degrade sRNAs associated with AGO2. Since PRMT5 is downregulated by bacterial infection, this regulation could potentially promote immunity by increasing AGO2 protein accumulation. As RG/GR repeats are also found in the N-terminal domain of other Arabidopsis AGOs such as AGO1, it will be interesting to investigate whether PRMT5-mediated arginine methylation also affects their stability.

## Conclusion

As can be grasped from this review, our current knowledge on how different proteases modulate the stability of RNA silencing components still remains limited. As indicated above, several endogenous E3 Ub ligases modulate the accumulation level of important players of the pathway, but we still do not understand how these proteins are recognized and ubiquitylated, which Ub chain topology is engaged, and how they are delivered to either the proteasome or the autophagy pathway. Interestingly, for some proteins, such as HYL1, AGO2, and AGO9, ubiquitylated peptides have been identified by proteomics analyses (Kim et al. 2013; Walton et al. 2016), but the E3 Ub ligases targeting them are still unknown (Table 1). It is likely that some of these silencing proteins could be targeted by multiple E3 Ub ligases that may act in different cellular compartments and/or in a tissue-specific manner. It will also be interesting to investigate how abiotic and biotic stresses control these proteolytic pathways to modulate both the production and the function of sRNAs in response to environmental cues. The degradation of HYL1, SGS3, and AGO2 caused by dark, heat stress, and the immune response, respectively, are already first examples of such regulatory mechanisms (Table 1), but many more are expected to be found. Finally, the most fascinating question to address is what could be the physiological function(s) of these proteolytic pathways? At present, this is very poorly understood. As discussed above, the degradation of unloaded AGO1 avoids the loading of illegitimate sRNA and thus off-target cleavage, which is harmful to mutant plants with reduced miRNA accumulation. However, the importance of this proteolytic mechanism in the context of a stress response, which would involve AGO1-RISC reprogramming, is still not known.

Remarkably, some recent reports in metazoans unraveled novel regulatory functions of E3 Ub ligases. For instance, it was found in mammals that ZSWIM8, a substrate receptor of a CRL3-type Ub ligase, is a key component of the target-directed miRNA degradation (TDMD) pathway, which occurs when a miRNA is associated with a target RNA with extensive complementarity (Han et al. 2020; Shi et al. 2020). In this case, conformational changes in AGO would be recognized by the ZSWIM8 Ub ligase resulting in the proteasomal turnover of the complex and subsequent degradation of the miRNA by cytoplasmic RNases. Another interesting study showed that fly and human AGO undergo LLPS on the ER allowing the recruitment of the E3 Ub ligase Ltnl to catalyze nascent peptide ubiquitylation to ensure efficient gene silencing (Gao et al. 2022). Whether, similarly, AGO proteins in plants serve as a platform for recruiting E3 Ub ligases in processes such as sRNA decay or protein quality control will deserve further studies.

## Data Availability

No new data were generated or analysed in support of this research.

## Dive Curated Terms

The following phenotypic, genotypic, and functional terms are of significance to the work described in this paper:

AGO2 Gramene: AT1G31280
AGO2 Araport: AT1G31280

SGS3 Gramene: AT5G23570
SGS3 Araport: AT5G23570

AGO4 Gramene: AT2G27040
AGO4 Araport: AT2G27040

DRB4 Gramene: AT3G62800
DRB4 Araport: AT3G62800

AGO5 Gramene: AT2G27880
AGO5 Araport: AT2G27880

DCL3 Gramene: AT3G43920
DCL3 Araport: AT3G43920

dcl4 Gramene: AT5G20320
dcl4 Araport: AT5G20320

DMS3 Gramene: AT3G49250
DMS3 Araport: AT3G49250

AGO6 Gramene: AT2G32940
AGO6 Araport: AT2G32940

CPL1 Gramene: AT4G21670
CPL1 Araport: AT4G21670

HYL1 Gramene: AT1G09700
HYL1 Araport: AT1G09700

COP1 Gramene: AT2G32950
COP1 Araport: AT2G32950

DCL1 Gramene: AT1G01040
DCL1 Araport: AT1G01040

RDR6 Gramene: AT3G49500
RDR6 Araport: AT3G49500

HCS1 Gramene: AT2G25710
HCS1 Araport: AT2G25710

cpl2 Gramene: AT5G01270
cpl2 Araport: AT5G01270

DCL2 Gramene: AT3G03300
DCL2 Araport: AT3G03300

HEN1 Gramene: AT4G20910
HEN1 Araport: AT4G20910

PAG1 Gramene: AT2G27020
PAG1 Araport: AT2G27020

PRL1 Gramene: AT4G15900
PRL1 Araport: AT4G15900

NRPD1a Gramene: A09p015000.1_BraROA
NRPD1a Araport: A09p015000.1_BraROA

AGO1 Gramene: AT1G48410
AGO1 Araport: AT1G48410

FREE1 Gramene: At1G20110
FREE1 Araport: At1G20110
